# ‘We saw she was in danger, but couldn’t do anything’: Missed opportunities and health worker disempowerment during birth care in rural Burkina Faso

**DOI:** 10.1186/s12884-016-1089-3

**Published:** 2016-09-29

**Authors:** Andrea Melberg, Abdoulaye Hama Diallo, Thorkild Tylleskär, Karen Marie Moland

**Affiliations:** 1 Centre for International Health, Department of Global Public Health and Primary Care, University of Bergen, PO Box 7804, N-5020 Bergen, Norway; 2 Centre MURAZ, Ministère de la Santé, 2054, Avenue Mamadou KONATE, 01 BP, Bobo-Dioulasso, Burkina Faso; 3 Department of Public Health, UFR-SDS, University of Ouagadougou, Ouagadougou, Burkina Faso; 4 Centre for Intervention Science in Maternal and Child Health (CISMAC), University of Bergen, Bergen, Norway

**Keywords:** Childbirth, Primary healthcare, Quality of healthcare, (Sub-Saharan) Africa

## Abstract

**Background:**

Facility-based births have been promoted as the main strategy to reduce maternal and neonatal death risks at global scale. To improve birth outcomes, it is critical that health facilities provide quality care. Using a framework to assess quality of care, this paper examines health workers’ perceptions about access to facility birth; the effectiveness of the care provided and obstacles to quality birth care in a rural area of Burkina Faso.

**Methods:**

A qualitative study was conducted in 2011 in the Banfora Region, Burkina Faso. Participant observations were carried out in four different health centres for a period of three months; more than 30 deliveries were observed. In-depth interviews were conducted with 12 frontline health workers providing birth care and with two staff of the local health district management team. Interview transcripts and field notes were analysed thematically.

**Results:**

Health workers in this rural area of Burkina Faso provided birth care in a context of limited financial resources, insufficient personnel and poorly equipped facilities; the quality of the birth care provided was severely compromised. Health workers tended to place the responsibility for poor quality of care on infrastructural limitations and patient behaviour, while our observational data also identified missed opportunities that would not demand additional resources throughout the process of care like early initiation of breastfeeding and skin-to-skin contact after birth. Health workers felt disempowered, having limited abilities to prevent and treat birth complications, and resorted to alternative and potentially harmful strategies.

**Conclusions:**

We found poor quality of care at birth, missed opportunities, and health worker disempowerment in rural health facilities of Banfora, Burkina Faso. There is an urgent need to provide health workers with the necessary tools to prevent and handle birth complications, and to ensure that existing low cost life-saving interventions in maternal and new-born health are appropriately used and integrated into the daily routines in maternity wards at all levels.

## Background

At the end of the Millennium Development Goal era, maternal and neonatal mortality rates remain unacceptably high in many countries in sub-Saharan Africa. In Burkina Faso, the lifetime risk of maternal death is one in 55, and about one in ten children will not survive their fifth birthday [1]. Whereas under five mortality has been declining during the last decades, neonatal mortality remains unchanged [2]. The time around birth is critical for both mothers and new-borns [3, 4]. Timely access to care at and around the time of birth is one of the main strategies to reduce maternal and neonatal morbidity and mortality [5, 6], as important is the quality of the care provided in health facilities [7]. Low quality facility birth care represents a missed opportunity to improve birth outcomes and increase the demand for facility birth care.

There is a unanimous call for improved quality care to prevent maternal and child deaths, but little consensus on how quality in healthcare should be defined [8]. Campbell et al. divide quality of care into *accessibility of services* and *effectiveness of the services provided* [9]*.* Accessibility is ensured when users can access the health structures and processes of care which they need. Effectiveness is divided into clinical and inter-personal care and involves different dimensions of the health system such as the structure or organisation of the services, the process of care, and the outcome of the care provided [9]. These dimensions are interlinked; for example, in order to achieve desired outcomes such as decreased infection rates in a facility, the availability of structures such as water and soap for hand washing is a prerequisite, but does not by itself ensure that birth attendants wash their hands.

Since most women and new-borns are well, and only some develop complications, quality of birth care should be considered differently from other areas of care provision [10]. As it is difficult to predict birth complications, it is an expressed goal in the global health community that all women should give birth assisted by skilled birth attendants [5]. This implies giving birth with a provider with midwifery skills, trained in the management of normal deliveries and the detection and management of complications during birth with the ability to refer to a higher level of care when needed [5]. At all primary health care facilities, basic emergency obstetric and neonatal care (BEmONC) should be made available to treat complications. This includes the possibility to carry out the following six key functions: providing parenteral antibiotics, anticonvulsants, oxytocic drugs, removal of placenta and retained products of conception, assisted vaginal delivery and resuscitation of new-borns [11]. Effective transportation systems to facilities with comprehensive care, including competence to carry out caesarean sections and blood transfusions, are essential for timely treatment of complications [12].

Given the numerous definitions of quality birth care, a variety of frameworks have been suggested to assess this outcome [8]. Many focus on outcomes such as case-fatality rate and caesarean section rate, some focus on structure such as equipment and personnel available, fewer focus on the process of care. One reason for this could be that the gold standard for assessing process is direct observations of the provision of care, which is costly and time-consuming [8]. The meaning of quality of care also depends on the assessor’s viewpoint. Users of health care would, for example, emphasize inter-personal aspects of care when evaluating the quality, whereas cost-effectiveness is a typical concern for managers [9, 13]. To gain knowledge about quality of care, the providers’ perspective plays an important role since they are situated at the point of service delivery and are able to technically evaluate the quality of clinical care more accurately than users of services.

In Burkina Faso, it is estimated that 66 % of births take place in a health facility with a skilled attendant [1]. Out of pocket costs for delivery care have previously been reported to impoverish patients and their families and to constitute an important access barrier [14]. A national subsidiary policy for deliveries and emergency obstetric care was implemented in 2006, subsidising 80 % of the costs of care and providing free emergency transportation [15]. Other reported barriers to facility birth have been distance to health facilities and women’s limited decision-making power within households [16].

The great majority of facility births in Burkina Faso take place in primary health centres (Centres de Santé et de Promotion Sociale). The data on outcomes of these facility births are suggestive of poor quality care; few are able to provide BEmONC and one prospective study found no difference in perinatal death risk between home-based and institutional deliveries [17, 18]. Studies of the quality of care provided in health centres have shown limited knowledge and compliance with guidelines among health personnel, unavailability of necessary drugs and diagnostic tests, delayed provision of care and inadequate counselling about danger signs during pregnancy and childbirth [18–21]. Even so, women giving birth in these health centres report a high degree of satisfaction with the services provided [22].

We conducted an exploratory qualitative study in four primary health care centres. In line with Campbell et al.’s framework we focused on access to care and the effectiveness of clinical and interpersonal care. We explored health workers’ perspectives on women’s access to facility birth and safe birthing, the strategies health workers employed to provide quality care; and what they experienced as obstacles to the provision of quality birth care.

## Methods

### Study site

The study was conducted in the Banfora and Mangodara health districts in the South-western part of Burkina Faso with an estimated population of around 500 000 inhabitants. Situated in West-Africa, Burkina Faso is among the world’s poorest countries, ranking 181^th^ of 187 on the Human Development Index 2011 [23]. In the study area, cotton production, subsistence farming and animal husbandry remain the main economic activities. With annual rainfalls of over 900 mm, the region of Banfora is amongst the most fertile and the least poor in the country [24]. Literacy is low in the region, 80 % of the adult population in the two health districts is considered illiterate. The main spoken language is Dioula; French is the official language, but is only spoken by those who have attended school.

The annual number of expected deliveries in the study area was 24 500 in 2011 [25]. At the time of the study, Banfora and Mangodara health districts had 39 primary health centres, usually with one dispensary and one maternity unit. Primary health centres referred women with obstetric emergencies to the regional referral hospital in Banfora town. The driving time from the health centres participating in the study to the regional hospital varied from five to 150 minutes. Not all health centres had access to an ambulance; some had to rely on private transportation.

### Data collection

The fieldwork lasted from September 2011 to January 2012 and the data collection took place in four primary health centres in the Banfora region, combining participatory observations and in-depth interviews.

As we assumed that working conditions would differ between urban and rural areas and depending on the monthly number of births, one urban, one semi-urban and two rural facilities were chosen. The number of health workers in the health centres varied from two to 12. The number of births per month varied from three to 100. The infrastructure of the health centres also varied substantially. Some had electricity and running water, while in others health workers had to rely on their personal torches as the only light source and on water from wells situated up to one kilometre from the health centre.

The two rural health centres were relatively large units situated approximately 65 km from the Banfora regional referral hospital. No smaller rural health centres were chosen due to practical concerns such as availability of housing and transport during data collection.

The first author, at the time a third-year medical student, carried out the participatory observations, both day and night for 12 weeks; three weeks in each of the four primary maternity units. The researcher was present at the health centres from two to eight hours every day, and during 14 night shifts. During this period, more than 30 deliveries were observed, 21 deliveries during daytime and 13 at night. The observations were non-structured; the researcher followed the health workers at work, asking questions and helping out with small tasks like getting the necessary drugs and equipment ready for the health workers. She did not work autonomously, nor did she provide direct patient care. Observations and reflections were noted daily in a field diary, providing information about health worker-patient interactions; health workers’ practices related to routine care such as pre- and postnatal consultations, reception and follow-up of women through first, second and third stage of labour as well as providers’ perspectives about working conditions, access to and quality of care.

In addition, the first author conducted 12 in-depth interviews with health workers providing obstetric care. Health workers were purposively selected for in-depth interviews on the basis of informal conversations and caregiving during observations in the health facilities, as well as their levels of experience and training, to represent different views. Two of the interviewees did not work in the study health centres, but were selected to represent the view of health workers in small rural health centres where, for practical reasons, observations could not be carried out. The 12 interviewees were two registered midwives, three registered nurses, one enrolled midwife, four auxiliary midwives, and two outreach health workers. Three of the interviewees were male. The recruitment of participants was ended at the point of data saturation when little new information emerged from the interviews. In addition, two medical doctors in the health district management team were interviewed about policy implementation at the centre level. The interviews included open-ended questions about access to facility pregnancy and birth care, the quality of care provided, working conditions, and health worker performance. All co-authors contributed to the making of the interview guide, which was piloted for its suitability in facilities not participating in the study, the interview guides were modified in the course of data collection based on observational data. The interviews were conducted in French in a separate room at the interviewees’ workplace, and lasted from 45 to 90 minutes. The interviews were recorded and transcribed verbatim.

### Data analysis

After initial analysis during fieldwork, interview transcripts and field notes were analysed thematically. NVivo 9 software was used to code and organize the data (http://www.qsrinternational.com). Firstly, after being familiarized with the datasets, initial codes were generated. These codes were grouped into categories and subsequently into themes. For instance, having a single blood pressure measurement device at the maternity ward was coded as *shortage of equipment*. This code was grouped with other codes to form the category *insufficient infrastructure as a barrier to routine care*. This, and others were then again grouped into the theme *Barriers to quality routine maternal and new-born care*. The combination of participant observations and interviews allowed for methodological triangulation, cross-checking the observational and interview data during analysis for improved validity [26].

### Ethical considerations

The study was approved by the national health research ethics committee of the Ministry of Health, Ouagadougou, Burkina Faso (Comité d’éthique pour la Recherche en Santé, CERS, No2011-9-57). Administrative clearance was granted by the regional health authorities in Banfora. Written informed consent was obtained from all interviewees. Verbal consent to participate at the care provision was granted by health workers for all observations. Health workers were asked to inform and ask all women in labour to consent to the researcher’s presence. To ensure the informants’ confidentiality, they are only referred to by their level of training throughout this paper.

## Results

We will firstly examine health workers’ perceptions about access to facility births and safe birthing. To explore health workers’ perceptions of the effectiveness of the healthcare provided, we will secondly explore aspects limiting quality of routine care, and thirdly the management of birth complications within primary health facilities.

### Access to facility births

#### Health facility as the only place to give birth

During IDIs and observations, health workers presented the health centre as the only safe and responsible place to give birth, and accused women not giving birth at the health centre of not being interested in ‘the best for their infants’, thus raising issues of responsibility. Some health workers claimed that home deliveries caused birth complications by the use of herbs and traditional medicine. Health workers reported that women and families arriving for vaccination after a home delivery were shameful, feared being scolded by health workers, and presented their excuses. Health workers emphasized that fundamental birth care such as the clean cutting of the cord, the timely detection of complications and the prevention of post partum haemorrhages by the routine administration of oxytocin during the third stage of labour could only be offered during facility births.

#### Reasons to give birth at home

Distance from the health centre was presented as the only acceptable reason to give birth at home. According to health workers, many women simply did not arrive in time, although some health workers suspected that this was due to the fact that women waited too long after the onset of labour to travel to the health centre.

‘The women like to blame the home births on the distance. We cannot argue against that. Some say it was late at night. Others say that the child arrived while they were preparing to go to the health centre. There are also some that give birth on the way to the health centre, who would like to come, but give birth on the way to the health centre. Well, but sometimes I think that these women do not get up early enough.’

Auxiliary midwife, IDI

Ignorance was perceived as the main driver for home births. According to health workers, it was more widespread for illiterate women or women living far away from the health centre to not yet understand the benefits of giving birth at a health centre. There was a general conception among health workers that informing the population at the health centre and in the communities to ‘make them understand’ the benefits of giving birth at the health centre was an important strategy for improving attendance rates.

Cost was not considered an issue since the introduction of the policy of subsidies for emergency maternal and neonatal care. All interviewees stated that the cost of a health centre birth was 900 FCFA (1.40 €), and that the population, with some few exceptions, were aware of the reduced price. Women coming to the primary maternity unit with complications after home births were observed not to benefit from the subsidy, but had to pay for the equipment and services provided. During interviews, health workers expressed how this policy was appropriate and justified considering the aim of increasing facility birth. They saw the reduced cost of facility delivery as an incentive that would make women give birth at the health facility, and informed women about it during antenatal care. In some health centres women giving birth at home were reported to receive emergency care for post-partum complication at reduced costs if she accessed care at health facility within 24 hours after birth. Otherwise she had to pay for the care provided. A health worker explained the practice in his health centre:

‘Anyhow, the EmONC-policy does not cover that. When they [women with complications after home birth] arrive, we prescribe everything and they pay at the drug store.’

*Auxiliary midwife, IDI*

Another important obstacle for facility birth, as perceived by health workers, was the limited privacy for women giving birth at the health centre. According to the interviewees, women tended to seek birth care at a late stage of labour to avoid neighbours from keeping track on the time spent at the maternity ward. According to health personnel, it was a sign of pride, especially in polygamous households, for a woman to endure the suffering and not seek help until the cutting of the cord. Additionally, at the health centre, neighbours would come to enquire about the progress of labour and get to know if the woman took a long time giving birth. Through the windows, neighbours and other patients could also hear the patients’ screams.

#### Perspectives on patient-provider interactions

Health workers expressed uncertainty when asked whether women were satisfied with the services offered at the health centre. They reported how women sometimes would give health workers blessings or small gifts to show satisfaction with the services provided. Otherwise, health workers found it difficult to know how women felt, as they did not show their dissatisfaction. How patients were received at the health centre was put forward as key to patient satisfaction, and as an area where health workers could improve their performance.

‘A patient, when she is received in a good way, she is already satisfied, she is already cured. But if you receive her badly, no matter what you do for her, it is nothing.’

Nurse, IDI

According to the study participants, the reception provided depended on the person on duty, which also was perceived to affect the attendance rate. It was seen as important to have good relations with local women and encourage them to come to the health centre to give birth. Some health workers emphasised the importance of being able to speak to the women in their local language to gain their trust. In many health centres women were reported to send relatives to see which health worker was on call before deciding the place of birth.

‘They come because, often the women chose a person. When it is the auxiliary midwife, the women come to confide. We had one auxiliary midwife here, she had been here for long, over five years. She was part of the village. The women came to give birth with her. When she was here and was on call, the women came. When other staff members were here, they went elsewhere… Often, they sent someone, asking who was on call before coming.’

Outreach health worker, IDI

### The delivery of routine maternal and new-born care

#### Health workers in primary maternity units

Health centres (CSPS) were commonly divided into a dispensary and a maternity unit in two different buildings. Larger rural maternity units as well as urban ones were most commonly headed by a midwife. Other birth attendants observed to work independently with outpatient consultation and birth care in the maternity wards were nurses, auxiliary midwives and outreach health workers. Midwives were recruited after they completed 13 years of school, and received a three-year training. Auxiliary midwives and outreach health workers were recruited after they completed primary school and attended a two-year training. Whereas midwives and auxiliary midwives’ curricula focused on maternal health care, outreach health workers curricula was reported by health workers to focus more on vaccination programmes and public health education. Some reported not having conducted a delivery before they were on duty alone and had to get assistance from the traditional birth attendant (TBA) in the village:

‘This was my first delivery in my first post. The woman arrived, she was my neighbour. She arrived, I did not know anything. Luckily for me, my luck was that it was 23.40 at night. I went to the village, where there was a traditional midwife. I looked for her, and she came. We did, she showed me how I should attend a birth. We attended the birth, and after that I have being attending births. I have not had any problem.’

*Outreach health worker, IDI*

Even though the expressed goal of the health district management during interviews was to have midwives in every health centre, this was not yet observed to be the case in small health centres where auxiliary midwives and outreach health workers constituted the majority of the maternity unit work force. Nurses typically worked in the dispensary unit during daytime, but assisted births when they were on call. Midwives and medical doctors did not, however, consider auxiliary midwifes and outreach health workers as able to ensure quality care at birth. One midwife characterized the auxiliary midwives curriculum as learning how to ‘to pull out and put down babies’, and argued that the auxiliary midwives curricula did not include the detection and management of birth complications. Members of the health authorities emphasized the need for continuous training for the large group of health workers working in the maternity units, but said that they did not have the capacity to supervise newly educated health workers. A medical doctor in the local health authorities explained:

‘Because the number of students in the schools, they are too many. The training capacities are not adapted to the number of students and that is what we see in the field. We ask ourselves where these people have been trained. But, what is done is done. They say you have got trained health workers, you have to do the best out of it.’

*Medical Doctor, IDI*

#### Limited compliance with clinical guidelines

In the maternity wards, several established standards of care as referred to by health workers during interviews were observed not to be followed. The researcher observed, with few exceptions, no skin-to-skin contact with babies after birth, limited surveillance during and after birth, and no hand washing between patients and procedures. These, and other national standards were communicated through the antenatal care booklet and posters produced by the Ministry of Health and displayed in the health centres. During interviews, non-compliance with clinical guidelines for routine maternal and new-born care was often mentioned, and explained by health workers by lack of time, inadequate infrastructures and everyday realities.

Health workers expressed that surveillance during labour competed with the delivery of routine care such as antenatal consultations, family planning consultations, immunization of infants and postnatal consultations as well as the surveillance of other women in labour. During nights, when routine care was not provided, health workers felt incapable of following the progress of labour with a partograph since they would be too exhausted for their routine tasks the following morning. Typically, at night, health workers were observed to ask the woman’s relatives to wake them up when delivery was approaching.

‘You see, she [the health worker] had five women in labour at once. Honestly, could she follow the process of labour correctly with partographs? It is difficult. One person assisting at five births cannot deliver quality birth care. You do whatever you manage.’

Midwife, IDI

In several health centres, health workers claimed that there were not enough beds, adequate lighting or toilet facilities for patients, which made post partum surveillance during the designated 72 hours difficult. Due to lack in health centre infrastructure, health workers therefore chose to let women return home shortly after birth; this was observed even one hour after delivery. During birth, the lack of running water, especially in rural units, was said to limit hand washing as well as the cleaning of equipment and the delivery room between patients. Some health centres were observed to be out of stock of several diagnostic tests, and were not able to provide urine tests to detect proteinuria (a sign of pre-eclampsia) and rapid HIV-tests during antenatal care. The researcher also observed a lack of smaller equipment, such as blood pressure measurement devices, scissors and foetoscopes, which also made the assurance of routine care difficult for health workers. In the urban health centre in the example below, blood pressure was only measured routinely during antenatal visits due to lack of equipment:

‘We have a huge problem with equipment. We have only one blood pressure measurement device here [maternity ward], as you have seen. One needs it for the family planning, one needs it for the antenatal consultation, one needs it to look after the women after birth and one needs it in the delivery room. It is complicated.’

Midwife, IDI

The antenatal care booklet indicated when a pregnant woman was having a high-risk delivery, and should be referred to the regional hospital in Banfora to give birth. There were different clinical indications for referral such as previous caesarean section, age, parity and blood pressure. Many women were reported not to follow the health workers’ advice due to financial constraints, and opted for a health centre delivery rather than going to the regional hospital. Primary health workers found some of the guidelines, such as age under 18 years at first delivery, as out of touch with local realities, and consequently were observed not to follow them.

‘Primipara younger than 18 years, they said should be referred. But if we should take that into account I am not sure if we would have any births at the health centre level. It is not sure. Because the majority of our primipara they are 16 years old, 17 years old.’

Auxiliary midwife, IDI

### Management of birth complications

Health workers found that birth complications such as postpartum bleedings, fresh stillbirths and delayed progress of labour were common, and related it to the women’s hard manual work in the fields until the time of delivery. From certain villages, health workers reported, women would only come to the health centre in case of a complication. Several health workers stated that a proportion of stillbirths and neonatal deaths were caused by the behaviour of women; either they got pregnant too often, or they refused to push when the foetus was showing sign of distress during labour. During interviews, health workers described how they would threaten the women with evacuations, caesarean sections, the possible death of the baby or with episiotomy by approaching scissors to the perineum in order to make the woman push and thus save the baby.

‘The women here are capricious, they refuse to push. Even when the amniotic liquid is coloured they refuse. I say push, or you will lose your baby. Or I tell them that they would have to go to Banfora [regional hospital]. Sometimes I even go to the consultation room to get the reference card. When I show them the reference card, they say to themselves that they do not want to go to Banfora, and they push better.’

Auxiliary midwife, IDI

#### Referral as first choice for health workers

All interviewees agreed on the fact that complicated deliveries should not be managed at the health centre level, but referred to the regional hospital in Banfora. At the health centre there was only a limited possibility to manage complications due to lack of competences and equipment. In many cases, families were reported to be unwilling to refer the woman because of the costs implied with the evacuation and the uncertain reception at the regional hospital. During observations outside the urban area of Banfora, the woman’s family was perceived to be responsible for finding a car or renting a minibus, and for paying the costs of the referral. Although health workers reported to be able to convince the family to go in most cases, some families decided to stay at the health centre against the health workers’ advice as exemplified below:

‘If you tell them they have to go to Banfora [regional hospital], some ask you to try to manage the situation here. I got the opportunity to ask a man why. He told me that he had accompanied his brother’s wife to Banfora, and after what he saw there, he preferred that his wife stayed here. We said no. It was a prolapse of the umbilical cord alive with heartbeats and a cephalic presentation, which we are instructed to refer. The husband said no, if the woman survives and only the child dies, it will not be a problem. That he prefers to stay at the health centre and that the woman gives birth, losing the child rather than going to Banfora. At that time, we did not have an ambulance. If he found the money to go to Banfora, the woman could still lose the baby on the way to Banfora. … They did not leave. The following day she gave birth to a fresh stillborn. When they were leaving, the husband kneeled, and thanked us. The woman also thanked us.‘

Auxiliary midwife, IDI

#### Delayed detection and transport

Health workers felt insecure about their own training and their ability to detect and handle obstetric emergencies when these occurred. One midwife heading a rural maternity unit claimed that the auxiliary midwives’ limited ability to examine the pelvis had several times lead to delayed identification of women in need for a caesarean section, with the consequence of foetal death. During observations of birth care, the partograph was not used a single time by health workers to monitor labour, regardless of level of training. Limited surveillance and knowledge about birth complications contributed to a delayed evacuation according to this auxiliary midwife working in a rural centre:

‘Sometimes, the woman arrives early in labour, but only afterwards you figure out that there is a complication. For example, once I received a woman here. She arrived around 1 am at night. She stayed until 1 pm, then I discovered that she was bleeding, even though she had not yet given birth. So, I called the chief nurse. He told me that the woman had signs of uterine rupture. We referred her. Unfortunately for us, she had a uterine rupture before arriving to Banfora. She arrived early, but we did not manage to detect the complication early enough.’

Auxiliary midwife, IDI

When having detected an emergency and a need for referral, the transport options were observed to vary a lot between health workers working in urban or rural health centres. Referrals in urban and semi urban areas were according to health authorities as well as health workers provided free of charge by the fire brigade (Sapeurs-Pompiers) with little waiting time. The rural health centres’ ambulances were observed to be in bad shape, and often did not function at all. Alternative means of transport were sought. Health workers conveyed that they advised women to leave on motorcycle or by bus. If the woman’s life was at risk, health workers stated that it was possible to call the regional hospital and get them to send an ambulance. The auxiliary midwife’s account of the woman with the uterine rupture continues:

‘The departure time causes problem. At that time the ambulance was out of order. We had to call Banfora [regional hospital], and then we had to wait.’

Auxiliary midwife, IDI

Health workers felt that they had limited abilities to manage complications while waiting for emergency transport. Even though they knew that it was not recommended, several health workers felt that they had no other options but pushing on the woman’s stomach with all their weight or giving oxytocin injections in cases of delayed labour. Such practices were also several times observed in the health facilities. Several interviewees reported how they felt disempowered, as they were forced to wait for emergency transport without being able to help the woman in any way, as exemplified in the same case as above:

‘We called the regional hospital, and they sent us an ambulance for her. We had to wait, we could not do anything. We saw that she was in danger, but couldn’t do anything. So we waited for the ambulance until 8 pm, for seven hours. We had no ambulance, what could we do?’

Auxiliary midwife*, IDI*

## Discussion

### Disempowerment of health workers

In resource poor settings, where comprehensive emergency obstetric and neonatal care is inaccessible, primary care is used as a strategy to provide birth care to all [8]. One of the main roles of primary care at birth is to detect and refer complications when these occur. The accounts of birth care providers in rural Burkina Faso reveal how health workers’ ability to assure timely detection and management of birth complications is severely limited. It has previously been documented that few of the primary health centres in Burkina Faso are capable of assuring BEmONC functions, especially assisted vaginal deliveries and removal of retained products [20]. In this study, health workers reported that women prefer staying in primary centres rather than being referred to the regional hospital for fear of unmanageable out-of-pocket costs. To prevent maternal and new-born morbidity and mortality and limit the number of referrals, all BEmONC services need to be provided at the health centre level.

Even though emergency transportation should be provided free of charge according to the subsidy policy, this was not the case in the study area. Access to emergency transport has also been a concern in other regions [22]. The combination of limited possibilities to manage complications at the health centre and little or no access to emergency transport made health workers into disempowered bystanders when life-threatening emergencies occurred. We argue that the despair of health workers faced with obstetric emergencies made them resort to alternative and potentially harmful strategies.

### Missed opportunities in the process of care

#### Clinical care

Previous studies have shown that users’ of institutional deliveries in Burkina Faso evaluate the clinical care provided as of good quality [22, 27], but patients have a limited ability to evaluate technical performance, and great discrepancies between reported and observed birth care have been shown [18]. Health workers in this study reported severe technical weaknesses in the surveillance of women in labour, routine hygiene and the management of complications. There was a continuous lack of material supplies, staffing and competences of staff within the health centres. These findings are not new nor unique for Burkina Faso [7, 28, 29], and are in line with the findings reported from the QUALMAT study conducted in the North-western part of the country [20, 30].

Health workers perceived several clinical standards and protocols available in the health centres as not relevant for their particular contexts, but as something you would do in an ‘ideal world’. This is in line with previous findings from Burkinabè primary health centres [30]. This non-compliance with set guidelines could be interpreted as a ‘know-do’ gap, but it may be more fruitful to discuss health workers’ ability to follow basic quality-promoting guidelines within a health system that is severely constrained in terms of both material and human resources. It has been shown that frontline health workers in Burkina Faso have limited access to clinical practice guidelines for maternal health, and that these are found to be of limited use [31]. Successful implementation of clinical guidelines depends on the guidelines themselves, their implementation as well as health worker, patient and environmental characteristics [32]. In a setting where health worker competences are seen as limited, there is an even larger need for clinical practical guidelines that are adapted to local realities.

It is important to note that certain aspects of sub-standard clinical care were not directly explained by insufficient infrastructure. Interventions that did not require additional costs such as skin-to-skin contact after birth to avoid hypothermia and early initiation of breastfeeding were not being routinely practiced in the health centres. Such low- or no-cost interventions constitute missed low-hanging fruits to substantially improve new-born health [18, 33]. In the study setting, we believe that the limited training of auxiliary midwives and outreach health workers practicing in the maternity units contribute to a limited knowledge of the importance of such interventions. In resource-poor settings such as the study area, there is a particular need for continuous training of health workers with a focus on interventions that do not require additional cost, time or resources.

#### Inter-personal care

Although health workers acknowledged that access to facility birth care depended on geographical factors, inter-personal care and structure of facilities, the health centre was presented as the only responsible place for a woman to give birth. Women who give birth at home were seen as less invested in the well-being of their babies. Similar attitudes among health personnel have been described elsewhere [34]. Such attitudes could be linked to the practice of blaming women for poor pregnancy outcomes [30]. These findings resonate with Douglas’ writings about risk and blame. According to her, risk is inevitably moral, and every poor outcome chargeable to someone’s account. This implies a ‘combination of moralistic condemning the victim and an opportunistic condemning [of] the victim’s incompetence’ [35].

The blaming of women for poor pregnancy outcomes can also be seen as a way for health workers to justify the mistreatment of women reported in this paper. Threats of poor outcomes for women and their babies, lack of confidentiality, neglect of women in labour, unwelcoming and poorly trained health workers, are all part of the larger problem of the mistreatment of women during labour [36]. It is evident that a health system deprived of resources may contribute to health worker behaviour, but it also seems reasonable to suggest that health workers utilize coercive methods deliberately to gain compliance from women, as reported from South-Africa [37].

Being blamed and mistreated for not using the services as prescribed by health workers, has implications for the utilisation of services and the overall trust in the health system [38]. The practice of sending a family member to find out who is on call, indicates that patients place their trust in individual health workers rather than in the health care institution, in this case the government health centre and its referral system [39]. In this setting, the high turnover of health workers may partly explain the problem of trust and may represent a barrier to the accessibility of care. Keeping health personnel in rural areas is a challenge for most countries’ health systems [40]. Although not explored in our findings, gifts of satisfaction to health workers have elsewhere been linked to the expectation of better treatment in the future, and thus interpreted as an element of bribery [41]. The practice of gift giving implies an additional cost for women and their families and is thus perceived as a threat to equal access to facility care. At the same time, such informal payments constitute an important source of income for health workers in low resource settings, and contribute to the retention of health workers [42]. In Burkina Faso, it has been shown that it is particularly hard to keep female health workers in rural areas because of lack of basic infrastructure such as water, electricity and schooling opportunities for their children [30].

### Methodological concerns

This study reports from four health centres in a rural part of Burkina Faso. Although substandard quality of care in primary health facilities in Burkina Faso previously has been documented [18, 19], the emphasis on frontline health workers’ and managers’ perspectives provides additional insight into the dynamics within primary health facilities providing birth care. Through observations and interviews with providers, we gained knowledge of providers’ perspectives on accessibility of services and the three components of health care effectiveness: the structure of care, the process of care and the outcomes of the care provided [9], and how these dimensions interact. This paper explored only providers’ perspectives; when users’ perspectives are presented, they are only seen through the lens of the providers of care.

As a young female student, the observer was perceived as a subordinate to the staff and was accepted in the ward, which facilitated participatory observation. However, social desirability bias may have influenced study participants both to describe and to perform best practices in the researcher’s presence. With no prior clinical experience from her home university and not having completed courses in obstetrics or paediatrics, she was only able to assess the health workers’ performance based on limited theoretical knowledge and nationally established guidelines as communicated to local health workers and was not able to provide advice on patient care. As the researcher experienced and developed an understanding of the practical constraints to the provision of quality care experienced by health workers, such as lack of water, electricity, referral possibilities and necessary drugs, her presence at the health centres may positively have influenced the interpretation of health worker actions in the findings. Staying for several weeks in each health centre, the health workers may have forgotten the observer’s role as a researcher and disclosed issues that they may not have revealed during formal interviews.

The observer did not understand the local language. This was a limitation when observing the patient/provider interaction and when the interaction between health workers took place in Dioula. When needed, the researcher asked health workers to explain to her in French what was happening.

The findings are limited to four health centres in the Banfora area and cannot be generalised beyond these study sites. However, the health centres in the study are subjected to the same health policy and the same health system culture and resource scarcity as health centres in other parts of Burkina Faso. Furthermore the services are provided in a socio-economic context with high levels of poverty and illiteracy which are not much different from other rural areas in the country. There is therefore reason to believe that the findings are relevant also in other rural health care settings in Burkina Faso.

## Conclusion

Quality of care as defined by Campbell et al. [9], which comprises both access to and effectiveness of the clinical and interpersonal care provided, was seriously compromised in the health centres in Banfora. The combination of limited abilities to manage birth complications and limited possibilities to refer women in need contributed to health worker disempowerment. Health workers tended to place the responsibility for poor quality of care on infrastructural limitations and to blame poor pregnancy outcomes on patient behaviour, while observation data also identified missed opportunities throughout the process of care that would not demand additional resources to address. There is an urgency to address the mistreatment of women during labour and to provide health workers with the necessary training both in midwifery skills and in respectful care. Basic infrastructure and the possibility to refer women to higher level of care are prerequisites to prevent and handle maternal and new-born complications. Implementation research is needed to guide action on how to ensure that low cost life-saving interventions such as skin-to-skin-contact after birth and early initiation of breastfeeding are employed at all levels of care.

## Abbreviations

BEmONC: Basic Emergency Obstetric and Neonatal Care
CSPS: Centre de Santé et de Promotion Sociale (Primary health centre)
HIV: Human Immunodeficiency Virus
IDI: In-depth Interview
